# From Here to There, Progenitor Cells and Stem Cells Are Everywhere in Lung Vascular Remodeling

**DOI:** 10.3389/fped.2016.00080

**Published:** 2016-08-17

**Authors:** Rebecca L. Heise, Patrick A. Link, Laszlo Farkas

**Affiliations:** ^1^Department of Biomedical Engineering, School of Engineering, Virginia Commonwealth University, Richmond, VA, USA; ^2^Department of Internal Medicine, Division of Pulmonary Disease and Critical Care Medicine, School of Medicine, Virginia Commonwealth University, Richmond, VA, USA

**Keywords:** stem cells, lung diseases, pulmonary circulation/physiology, bioengineering, mechanotransduction, cellular

## Abstract

The field of stem cell biology, cell therapy, and regenerative medicine has expanded almost exponentially, in the last decade. Clinical trials are evaluating the potential therapeutic use of stem cells in many adult and pediatric lung diseases with vascular component, such as bronchopulmonary dysplasia (BPD), chronic obstructive pulmonary disease (COPD), idiopathic pulmonary fibrosis (IPF), or pulmonary arterial hypertension (PAH). Extensive research activity is exploring the lung resident and circulating progenitor cells and their contribution to vascular complications of chronic lung diseases, and researchers hope to use resident or circulating stem/progenitor cells to treat chronic lung diseases and their vascular complications. It is becoming more and more clear that progress in mechanobiology will help to understand the various influences of physical forces and extracellular matrix composition on the phenotype and features of the progenitor cells and stem cells. The current review provides an overview of current concepts in the field.

## Introduction

Chronic lung diseases are among the most vicious killers in children and adults. With a growing body of evidence based on ongoing research efforts, we are beginning more and more to understand the true importance of stem and progenitor cells: that these cells are suitable for use in cell therapies and in regenerative medicine, but even more that lung resident and even circulating or bone marrow (BM)-derived stem progenitor cells may be important culprits in many disease processes itself (1, 2).

**Figure 1:**
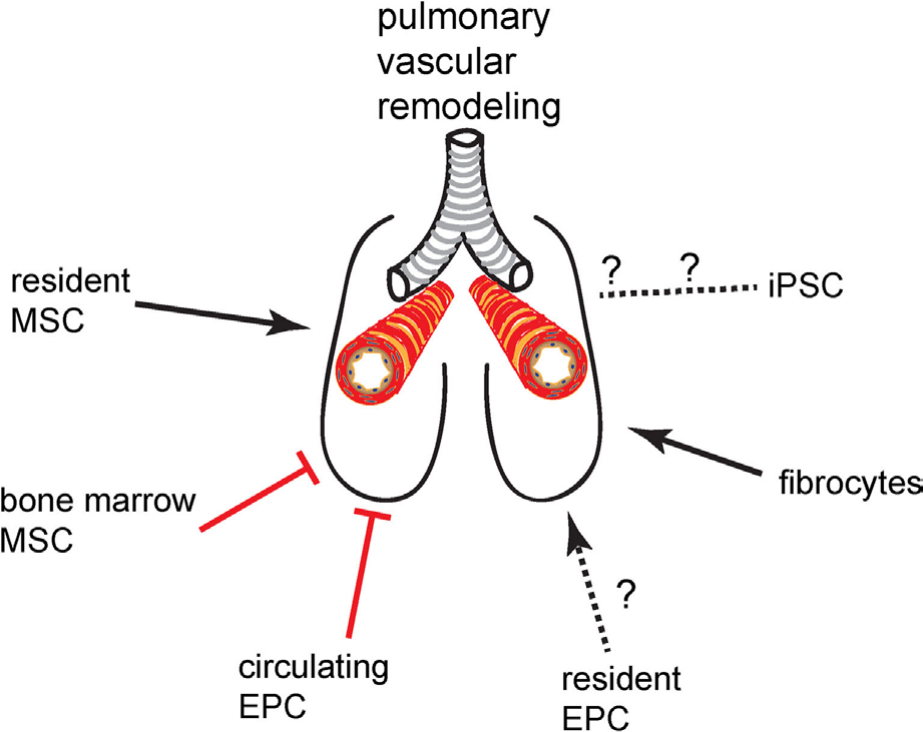
**Promoting or inhibitory effect of progenitor cell and stem cell populations on pulmonary vascular remodeling**.

There are a limited number of stem and progenitor cell populations that are currently of interest in vascular remodeling and cell therapy in the lung. These cells and their putative net effect on pulmonary vascular remodeling (promoting vs. inhibitory) are given in Figure 1. Here, we provide a short introduction on the most important features of these cells that are pertinent to lung diseases with vascular complications. References to more detailed articles on these cells are provided in the bibliography of this article.

### Mesenchymal Stem Cells

Mesenchymal stem cells (MSCs) are among the best investigated stem cell populations (3). BM-derived MSCs are the most frequently studied MSCs. The international society for cell therapies has set minimum requirements for the definition of MSC: positive for surface markers CD73, CD90, and CD105 and negative for CD14, CD34, and CD45 (4). In addition, MSCs also need to be able to generate adipocytes, osteoblasts, and chondroblasts in lineage differentiation assays, *in vitro* (4). This criterion shows the multipotency of these cells, meaning that MSCs are able to give rise to several cell types within the mesenchymal lineage. MSCs have a typical fibroblast-like morphology when cultured *in vitro* and grow in colony-forming units-fibroblast-like (CFU-F) when seeded in limiting dilution (5). Because of their lack of human leukocyte antigen (HLA) expression, the immune system is largely blind toward MSC, leading to what has been called the “immune-privileged status” of MSC (6). This immune-privileged status has made the MSC an ideal candidate cell for transplantation approaches, as HLA incompatibility is not an issue with MSC. BM-MSCs have shown great promise for cell therapy in various animal models of lung disease and undergone testing for safety and efficacy in several patient groups of lung diseases as will be outlined in the following paragraphs.

In addition to BM-MSCs, lung resident MSCs or mesenchymal stromal progenitors have raised attention due to their potential contribution to several disease processes (7–12). These cells are less clearly defined in their phenotype, although authors use many of the criteria that define BM-MSC.

#### Bronchopulmonary Dysplasia

Preterm infants who are treated for postnatal respiratory distress are at risk to develop bronchopulmonary dysplasia (BPD) as a consequence of mechanical ventilation and oxygen therapy (13). BPD pathology shows elements of inflammation, abnormal alveolarization, fibrosis, and pathological vascular remodeling (14). The histopathological correlates of the vascular remodeling are dysmorphic capillaries in the interior of thickened alveolar septa, as well as periarteriolar thickening, degeneration of elastic laminae, and increased thickness of the vascular smooth muscle layer (15, 16). There is a mixed bag kind of literature found for the role of MSC in BPD, and it is not clear whether MSCs are friend or foe in BPD (17). In one study, the presence of MSCs in tracheal aspirate predicted the development of BPD and was associated with increased mortality (18). In animal models, BM-MSCs successfully improved neonatal lung injury and arrested alveolar growth (19, 20). It is even more interesting that MSCs rely on their secretome for their beneficial effect, which supports the concept that MSCs protect cells not by direct replacement of cells but rather by paracrine effects (21).

#### Chronic Obstructive Pulmonary Disease

Patients with chronic obstructive pulmonary disease (COPD) present with a diverse variety of possible phenotype ranging from emphysema to chronic obstructive bronchitis, all characterized by irreversible airflow limitation. The lung vasculature is also affected by a chronic inflammatory response in the lung: This notion is supported by the findings that, for example, the pulmonary arteries show adventitial infiltrates with CD8^+^ T lymphocytes and cigarette smoke promotes accumulation of neutrophil granulocytes in the lung capillaries (22, 23). Pathological findings in the lung vessels of patients with COPD include increased wall thickness, changes in the composition of the extracellular matrix (ECM) causing stiffening of the blood vessel wall, and increased vascularization of the bronchial wall (23–25). Because COPD belongs to the top killers, efforts are underway to evaluate the role of MSC in the natural course of the disease and as a means for regenerative medicine to revert the severe tissue destruction found in the lungs of COPD patients. Some concepts suggest that aging of BM-MSCs could contribute to the development of COPD, e.g., through stem cell depletion (26). Transplantation of BM-MSCs is being considered for therapy of patients with COPD: a recent study has demonstrated the safety of giving BM-MSCs to patients with COPD (27).

#### Pulmonary Arterial Hypertension

The changes in the pulmonary arteries in pulmonary arterial hypertension (PAH) range from thickening of the smooth muscle layer and distal extension of a smooth muscle layer to non-muscularized precapillary arterioles to complex multicellular concentric and plexiform lesions (28–31). The literature regarding MSC in pulmonary vascular disease are ambivalent: multipotent MSCs are found in vascular lesions of patients with thromboembolic pulmonary hypertension (PH) (12) and pericytes, mesenchymal vascular cells that are a source of MSCs in the lung, contribute to pulmonary artery remodeling in PAH (11). On the other hand, several studies have demonstrated that MSC transplantation reduces PAH in experimental animal models (32–37). There is a potential difference between BM-derived MSCs that are used for cell transplantation and therapy and lung resident MSCs that may be part of the remodeling process in the vasculature. Future investigations are not only important to answer this question but may also yield a better understanding of the interaction between lung resident and circulating BM-derived cell populations in PAH pathobiology.

#### Idiopathic Pulmonary Fibrosis

The understanding of the disease pathobiology has come a long way in idiopathic pulmonary fibrosis (IPF). In its humble beginnings, IPF was understood as a disease of dysregulated fibroblast proliferation and activity (38, 39). Pathobiological concepts circled around the so-called “fibroblastic foci,” areas of active fibroblast growth and deposition of ECM with typical sub-epithelial localization. With the onset of finer methods and a better understanding for cell plasticity, the concept that IPF likely starts from repetitive microinjuries to the alveolar epithelium, causing epithelial apoptosis and activation of myofibroblasts arose (40). We have shown that endothelial cell (EC) apoptosis also occurs in areas of active fibrosis both in IPF and in experimental PF (41, 42). Other studies have also found heterogeneous remodeling of the lung vasculature in IPF, with reduced vascular density and aberrant capillaries in regions of active fibrosis and elevated vascular density in border areas adjacent to fibrotic areas (43–45). Based on data showing that PH in IPF reduces survival, it is likely that lung vascular remodeling is more than just a bystander in this devastating disease (41). Various sources of the myofibroblasts, which are the cornerstones of fibrogenesis, have been discussed over time, including resident fibroblasts, epithelial cells *via* epithelial-to-mesenchymal transition (EMT), circulating mesenchymal progenitors (“fibrocytes”), endothelial cells *via* endothelial-to-mesenchymal (EndMT) transition, and pericytes (9, 10, 46–56). Recent work has demonstrated that resident mesenchymal cells, particularly pericytes, give rise to the vast majority of myofibroblasts (9, 10, 56). This is quite interesting because at least a fraction of the pericytes likely originates from multipotent mesenchymal progenitor/stem cells (57, 58). Hence, these results suggest that lung-resident MSCs contribute to the disease process. It is interesting that current therapeutic trials evaluate BM-derived MSCs as potential therapeutic target and that the results so far are promising. These studies show, so far, that it is safe to treat IPF patients with BM-MSCs, an outcome that may be counterintuitive at first (59–61). But the results make sense if MSCs are seen as anti-inflammatory cells that support repair processes mainly *via* paracrine secretion, as suggested by preclinical studies, yielding exciting results by transferring only exosomes of MSCs (62, 63).

### Fibrocytes

Fibrocytes have been discovered as a population of hematopoietic cells with ability to produce collagen and to differentiate to myofibroblasts (64). Although shown to accumulate in multiple organ systems during injury repair and fibrosis, fibrocytes have received particular attention in the field of chronic lung diseases, because of a possible contribution of fibrocytes to disease development and progression: such diseases include *ILD* of the adult, such as *IPF*, where high levels of circulating fibrocytes have been demonstrated to indicate a poor prognosis (48). In addition, histological evidence for fibrocytes is present in lung tissue from IPF patients (46). Homing of fibrocytes to fibrotic lung tissue depends upon CXC chemokine ligand 12 (CXCL12), a finding that provides an interesting option to block tissue accumulation of fibrocytes as a potential therapeutic avenue (65). Fibrocytes may also represent a target of therapy in *PAH*, as evidenced by reduced fibrocyte accumulation as a response to treprostinil therapy in an animal model (66).

### Endothelial Progenitor Cells

Different cell entities have been summarized under the term “EPC”: EPC have been recently shown as resident cells in the lung circulation and initially as circulating cells (67–71). There are two main ways to identify circulating EPC: first, EPC can be detected within blood mononuclear cells by flow cytometry with specific surface markers (72). The most frequently used marker combination is CD34^+^ CD133^+^ VEGFR2^+^. The identity of these cells as “true” EPC has been controversial for quite a while (68, 73). Second, EPC can be detected by growth: depending on culture principles, early outgrowth CFU-Hills have been distinguished from late outgrowth endothelial colony-forming cells (ECFC) (71, 74, 75). Today, the consensus is that CFU-Hills are rather mononuclear cells with endothelial markers that exert protective effects on endothelial cells largely in a paracrine fashion (76), whereas ECFC are “true” EPC with the ability to replace damaged EC by engraftment (74). Different results have been obtained in preclinical studies for *PAH*, largely depending on the type of cell that the investigators used: Whereas endogenous EPC seem to be functionally impaired with higher than control proliferation rate but lacking ability for angiogenesis (72), therapy with genetically modified EPC has been successful for treatment in animal models of PAH (77). A clinical trial to determine whether genetically modified EPC have the same therapeutic benefit on PAH patients failed to provide the expected results (78).

In addition to circulating EPC, there is evidence for several EPC populations in the adult lung: Alphonse et al. have shown that ECFC can be isolated from the lung periphery and that these ECFC are functionally impaired in hyperoxia lung injury (79, 80). There is also evidence for EPC with high ability for self-renewal in the adult murine lung (81). These cells have been named “vascular endothelial stem cells” and represent EPC that are on the very top of the lung EC hierarchy. The finding of these endothelial stem cells in the blood vessels of the lung is consistent with previous reports of systemic blood vessels harboring a complete hierarchy of EPC (82). EPC at various levels of self-renewal and differentiation could indeed provide a basis for extensive vascular regeneration. On the other hand, because of the high ability to grow, some of these EPC or endothelial stem cells may also contribute to generate the apoptosis-resistant EC that are found in the complex vascular lesions of PAH patients (83). Evidence for mono- and polyclonal EC proliferation in complex lesions of pulmonary arteries in PAH indeed suggests a contribution of EPC or endothelial stem cells to the development of plexiform lesions in PAH (84). Much work needs to be done to characterize the EC/EPC/endothelial stem cell hierarchy in the lung and to identify the potential of EPC/EC stem cells for regenerative medicine or disease pathobiology.

### Induced Pluripotent Stem Cells

Adult stem cells are mostly understood as multipotent cells, meaning that adult stem cells can give rise to multiple cell types within a given lineage (85). The classical example for a multipotent stem cell is the MSC with the ability to produce cells of multiple skeletal cell types, such as osteoblasts, chrondroblasts, adipocytes, and muscle cells (3, 86). In contrast, during embryonic development, there is an early population of stem cells that have the ability to make cell lineages from all three germinal layers (87). This ability is called pluripotency, and the cells are described as embryonic stem cells. No single marker or transcription factor can define pluripotency, but instead a panel of functional assays in combination with activity of several transcription factors, e.g., Oct-4, Nanog, Klf4, and others, can help to identify pluripotent cells (87–90). In recent years, a variety of strategies have been shown to use a mature cell, such as fibroblasts, and induce pluripotency in such a cell. The strategies range from simultaneous overexpression of several transcription factors to chemical stimulation methods (88–94). So why is it an advantage to generate such iPSC? First, pluripotent cells are very powerful cells that can give rise to almost any cell lineage in the body and, therefore, their value for regenerative medicine may be skyrocketing. But human embryonic stem cells, which are the “natural” pluripotent stem cells, are highly restricted and difficult to obtain. Hence, iPSC would provide a much easier accessible and less regulated source of pluripotent stem cells. Second, iPSC allow generating pluripotent stem cells from tissues such as skin of patients and, therefore, providing a patient-specific and genetically identical source of cells. Such approaches will likely be very useful in the dawning age of “personalized medicine.” Recent work in the vascular field has demonstrated that iPSC can give rise to different vascular lineages, including EC (95, 96). The ability to generate genetically identical EC from, e.g., skin fibroblasts will provide a unique tool for regenerative medicine, including seeding of decellularized lung matrices with genetically engineered but patient-specific lung cells as prerequisite for using these recellularized lungs as transplant organs (97). Despite all the enthusiasm and hopes that flare up with such exciting and groundbreaking progress, these so powerful iPSC also need to be careful evaluated for adverse effects when transplanted to patients and seeded in organ matrices to generate future transplant organs: because iPSC can give rise to so many different lineages and have a high self-renewal potential, iPSC harbor the danger of giving rise to produce genetic and epigenetic changes, immunogenicity, and tumor cells (98). Even the possibility of such adverse effect of iPSC therapy needs to be carefully considered, and the risks have to be carefully weighted against the clear and exciting potential to benefit the patient by using iPSC.

### Role of Biomechanics and Stem Cell Mechanobiology in Vascular Remodeling

As science progresses with a better understanding of the role of the various stem or progenitor cell types in contribution to lung disease, repair, or regeneration, the mechanical microenvironment has emerged as a major player. Mechanical signal transduction through focal adhesions, the glycocalyx, mechanically activated ion channels, primary cilia, and force across cell–cell junctions may all play a role in the contribution of stem cells to pulmonary vascular remodeling (99). The dynamic mechanical environment of the lung lends itself to study the effects of mechanobiology in normal physiology and pathology of the lung microvasculature. The mechanical environment is essential to stem cell behavior during lung development and during pathological vascular remodeling events, which occur in *BPD, IPF, COPD*, and *PAH*.

During development of the pulmonary microvasculature, the fetal lung undergoes hydrostatic pressure between 1–2 mmHg, which maintains the lung in a distended state (100). Fetal breathing movements cause cyclic mechanical loading to the developing alveoli and microvasculature causing shear stress (101). Following the development of the primitive circulatory system, blood velocity and shear stress is critical for embryonic vascular patterning, remodeling, and proper development (102). Lack of proper mechanotransduction at any stage in the developmental process can lead to structural abnormalities and an underdeveloped lung.

*Bronchopulmonary dysplasia* is characterized by impaired alveolarization and vascularization and often occurs following mechanical ventilation. The impaired structure of the lung microvasculature leads to abnormal fluid flow and reduced gas exchange. High air flow from mechanical ventilation can increase lung injury, leading to the development of BPD (103).

*Idiopathic pulmonary fibrosis* is associated with progressive fibrotic scarring and stiffening of the lung ECM (104). This scarring is also associated with loss of compliance. The overall stiffness of the alveolar septa is increased by two- to threefold as measured by atomic force microscopy (105). IPF is also associated with increased vascular remodeling and may be associated with increased PH.

In *PAH*, pulmonary vascular remodeling is influenced by both hemodynamic and ECM changes. By its very nature, the remodeling in PAH causes increases in the pulmonary vasculature pressure as shown by animal models of severe PAH (106). But increased pulmonary artery pressure, e.g., by vasoconstriction, may also contribute to remodeling of pulmonary arteries and even foster the development of complex lesions in pulmonary arteries (107, 108). Furthermore, the ECM changes that occur in remodeling lend themselves to increased substrate stiffness (109, 110).

*Chronic obstructive pulmonary disease* also causes changes in lung mechanics and vascular remodeling. A reduction of vascularity in the alveolar septa occurs in emphysema (111). Loss of alveoli causes lung hyperinflation and overdistension of the remaining alveoli and capillaries. Furthermore, pulmonary arterial stiffness and blood pressure are increased in COPD patients and are not reduced with pulmonary rehabilitation (112).

These altered mechanical environments and their effects on stem cell contribution to pulmonary vascular remodeling warrants further study. The general physiological response of stem cells to mechanical signaling has been studied greatly *in vitro*, with *in vivo* studies largely lacking. Furthermore, specific studies of the mechanical environment on stem cells residing in the lung vasculature are especially absent. Information may be gleaned by understanding stem and progenitor cell response to general mechanical conditions. We will briefly review the state of the science of stem cell response to each of three mechanical conditions: shear stress, cyclic strain, and substrate stiffness as they may pertain to pulmonary vascular remodeling and stem cell-based therapies. These mechanical loading conditions and their effect on cell signaling and phenotype are summarized in Table 1.

**Table 1:** **Mechanical loading conditions and their effect on cell signaling and phenotype**.

Cell type	Loading condition	Increased	Reference
EPC/VESC	Shear stress	VEGF-R	(81, 113–115)
		E-CAD	
		Tyrosine-kinase-1	
		Ephrin B2	
		Notch 1/3	
		Hey 1/2	
		Activin-RLK	
	Stiffness	Tube morphogenesis (required VEGF), and increased cell adhesion on stiffer substrates	(116, 117)
Bone marrow-derived progenitor cells	Cyclic strain	α-SMA	(118)
		h1-calponin	
Epithelial	Stiffness	EMT on harder surfaces; apoptosis on softer substrates	(119, 120)
Endothelial	Stiffness	Increased proliferation with increased stiffness; inhibited EndMT with decreased stiffness	(121–123)
	Cyclic strain	Damage and inflammation	(124, 125)

#### Shear Stress

Whether *in vitro* or *in vivo*, cells generate force and are often exposed to force, and both can influence stem cell fates (126). Exposed forces can be axial or shear in nature, as one would find in fluid flow (115). Embryonic stem cells and BM-derived EPC have been shown to differentiate into endothelial cells (113). In fact, under flow conditions, EPC can form functional blood vessels from single c-kit^+^ cells (81). Human EPC demonstrate increased proliferation and VEGF-receptors, endothelial cadherin, and tyrosine-kinase-1 under laminar flow conditions up to 2.5 dyn/cm^2^ (115). Furthermore, under laminar flow conditions, artery phenotypes of Ephrin B2, Notch 1/3, Hey 1/2, and activin receptor-like kinase increased in EPCs (114). The altered signaling found in these studies under fluid shear stress emphasize the importance to examine stem cell response under dynamic mechanical conditions, such as those found in the diseased lung.

#### Cyclic Mechanical Strain

A body of work has been examined wherein microvascular endothelial cells exposed to cyclic mechanical loading have been shown to contribute to damage and inflammation (124, 125). However, there has been little work, to date, examining the role of stem cells in this process. Under cyclic strain, EPC may differentiate into smooth muscle cells, since it has been shown that platelet-derived growth factor-BB can induce EPC differentiation into smooth muscle cells (127). BM-derived progenitor cells exposed to cyclic stretch increased α-smooth muscle actin and h1-calponin (118).

#### Altered Substrate Stiffness

Vascular remodeling alters ECM composition and stiffness. It is well known that naive MSCs can be induced to specific lineages using soluble induction factors in conjunction with tissue-specific substrate stiffness (128). Mechanical stiffness has been shown to increase differentiation of BM-derived mononuclear cells into endothelial progenitor cells (129). Tube morphogenesis from EPC required the combined effects of stiffness and VEGF (116). Differentiation of EPC based on stiffness alone has not been well established. One group looking at ECFC found different cell adhesion attributes based on stiffness but found no phenotypic changes associated with stiffness (117). However, this experiment utilized media that may have prevented differentiation. Transdifferentiation, or lineage deprogramming, can also occur through alterations of the substrate stiffness. In epithelial cells, spontaneous EMT can occur on stiffer substrates (119). In murine breast cancer, stiff substrates can induce EMT while softer substrates induce apoptosis (120). For endothelial cells, TGF-β1 and TGF-β2 play the most important role to induce EndMT, but stiffness can significantly impact EndMT, with softer substrates almost inhibiting the effects of TGF-β (122, 123). Substrate stiffness does play a major role in cellular adhesion, proliferation, morphology, and migration (130). While forces may induce or prohibit differentiation, the effects are much more significant with growth factor support. Further studies are necessary to relate these *in vitro* studies to the *in vivo* effects of vascular remodeling *in vivo*.

In conclusion, stem cell contribution to vascular remodeling in lung pathologies will be dependent on the mechanical environment. These changes will play a role in cellular differentiation and matrix deposition, all contributing to the pathological process. In stem cell therapy approaches, these effects will need to be harnessed to promote positive tissue remodeling and regeneration.

## Author Contributions

Conception of work: RH, PL, LF. Drafting or revising the manuscript: RH, PL, LF. Final approval: RH, PL, LF. Agreement to be accountable for all aspects of the work: RH, PL, LF.

## Conflict of Interest Statement

The authors declare that the research was conducted in the absence of any commercial or financial relationships that could be construed as a potential conflict of interest.
